# Loneliness of Older Immigrant Groups in Canada: Effects of Ethnic-Cultural Background

**DOI:** 10.1007/s10823-015-9265-x

**Published:** 2015-05-16

**Authors:** Jenny De Jong Gierveld, Suzan Van der Pas, Norah Keating

**Affiliations:** Netherlands Interdisciplinary Demographic Institute (NIDI), P.O. Box 11650, 2502 AR The Hague, The Netherlands; Department of Epidemiology and Biostatistics, EMGO Institute for Health and Care Research, VU University Medical Center, Amsterdam, The Netherlands; Department of Human Ecology, University of Alberta, Edmonton, Canada; Centre for Innovative Ageing, Swansea University, Swansea, UK; Africa Unit for Transdisciplinary Health Research (AUTHeR), North-West University, Potchefstroom, South Africa

**Keywords:** Canada, Ethnic background, Immigrants, Loneliness, Older adults

## Abstract

This study aimed to explore the loneliness of several groups of older immigrants in Canada compared to native-born older adults. Data from the Canadian General Social Survey, Cycle 22 (N older adults = 3,692) were used. The dependent variable is the 6 item De Jong Gierveld loneliness scale. Determinants of loneliness included country of birth, ethnic background (cultural context); belongingness (community context) and social networks (social context). Results showed that only some immigrant groups are significantly lonelier than older adults born in Canada. Immigrants with similar language and culture are not lonelier; while those from countries that differ in native language/culture are significantly higher on loneliness. Multivariate analyses showed the importance of cultural background, of composition of the network of relatives and friends, and of local participation and feelings of belonging to the Canadian society in explaining loneliness of older immigrants.

## Introduction

“In aging, we long for what we have left behind. I call this aging in a foreign land” (Kalache 2013).

In an increasingly global world, the experience of aging in a foreign land is part of the late-life experience of many older adults. Kalache’s statement is especially relevant in countries that have long histories of immigration. Canada is one such country. Since the 19^th^ century, it has been one of the main receiving countries, yearly welcoming thousands of immigrants (George 2006). The proportion of immigrants in Canada who are aged 65 years and older has increased over the decades, from less than 6 % in 1921 to 18 % in 1996 (Boyd and Vickers 2000), and almost 20 % in 2011 (Statistics Canada 2011a, b). A striking feature of Canada’s older population is that immigrants now comprise 30 % of all Canadians aged 65+.

International migration is a salient life course transition that may influence trajectories of connections to family, friends and communities (Treas and Batlova 2009). Migration can affect the likelihood of continuity of relationships with members of one’s kin network and the potential for optimizing and diversifying social contacts in the new environment (Ajrouch et al. 2005). Developing friendships in a new country, especially friendships outside the circle of ones’ own ethnic group may be challenging, especially if one lacks fluency in the language of the receiving country (Wong et al. 2005). Further, while engagement in community activities and organizations are seen as a core element of active aging (Walker 2009), the intersectionalities of immigrant status, language/culture can result in the exclusion of some older persons (Keating and Scharf 2012; Viruell-Fuentes et al. 2012).

Given the high proportion of older adults in Canada who are immigrants, it seems important to better understand how immigration might influence the connectedness of migrants to people and communities as they navigate the latter part of their lives. In this study we address this question through examining factors associated with loneliness for immigrants and native born older persons.

### International Migration and Loneliness

International migration is a process that encompasses many challenges and uncertainties related to adapting to new living and working circumstances. The impetus for international migration may emanate from economic factors including better employment opportunities; from political factors such as wars or systematic discrimination in the sending country; or from family reunification. Each may differentially influence potential for adaptation to the new environment (Castles et al. 2014). Moreover, the decision to start international migration may not be that of an individual migrant but a broader household strategy to improve well-being, increase income, and raise investment capital of all household members (De Haas and Fokkema 2010).

Among the many challenges facing immigrants is the creation of a sense of place in the new country (Lewis 2009). Developing satisfying relationships with others is an important component of becoming grounded in a new place. People carry with them expectations and standards regarding the size, composition and functioning of their networks (De Jong Gierveld 1987). Meeting these expectations with new people in a new setting cannot be assumed but depends on cultural background as well as personal characteristics and environmental contexts. We are just beginning to tease out the relative importance of these characteristics and contexts in influencing later-life connectedness and loneliness of immigrants. For example, in a recent study of older Canadians, Wu and Penning (2015) found that immigrants have higher levels of loneliness than native-born Canadians, supporting the contention that immigrant status is important in understanding late-life loneliness. Importantly, they call for better understanding of the diversity among older adults thus avoiding assumptions that immigration per se is a risk factor for loneliness.

Loneliness has been defined as an unpleasant experience resulting from a person’s evaluation that their network of social relations is inadequate in either its’ quantity or quality (De Jong Gierveld 1987; Perlman and Peplau 1981). An important element of this definition is that it is grounded in the experience of the individual. There is no optimal size or set of relationships in a social network. Rather, loneliness occurs when individuals’ evaluation of their network falls short of their wishes for the network.

Loneliness of older adults has become an issue of considerable international concern because of its links to poor health, negative changes in living circumstances and social exclusion (Newall et al. 2014; Scharf and Keating 2012). Its prevalence among older people in the UK has prompted a national campaign to reduce its incidence and negative effects (Campaign to End Loneliness 2011). There is a growing body of evidence that rates of loneliness differ across countries, in part because of cultural standards related to expectations of kinship and friendship relationships (e.g., Van Tilburg et al. 2004). Yet to our knowledge there has been no exploration of within-country variations in loneliness that might arise from similar diversities.

### Conceptual Framework

An ecological model is used to frame the analysis of the importance of cultural background, community connections and social networks in determining the risks of loneliness of older adults. Bronfenbrenner’s (1994) classic approach allows for conceptualizing these influences as micro, meso and macro in terms of their immediacy to the person. For this study, these sets of influences are augmented by a critical human ecology approach (Keating and Phillips 2008) in which we challenge the perception that immigrants as a group are lonelier than native born older persons by exploring how diversity of immigrant cultural background relates to loneliness in later life.

#### Micro Level

At the micro level, the social context of kin and non-kin relationships is important in influencing loneliness. Researchers have found that the size, composition and support exchanges within the social network are strongly associated with levels of loneliness among older adults (Pinquart 2003). Family relationships are important elements of these networks. Adult children are a source of companionship, closeness, and sharing, particularly for those who live alone (Dykstra 1993; Pinquart 2003). Moreover, sibling support has been found to buffer against loneliness of older adults (Voorpostel and Van der Lippe 2007). The importance of friends, colleagues and acquaintances for alleviating loneliness also is well documented (Blieszner and Adams 1992; Cacioppo and Patrick 2009). Best friends can step in and function as confidants and in doing so help alleviate loneliness, in particular for never-partnered or childless older adults (Dykstra 1993; Pinquart 2003).

Perceived quality of the social network has been shown to be more important in explaining levels of loneliness than the size of the social network per se (Perlman and Peplau 1981; Victor et al. 2000). Routasalo et al. (2006) found that unfulfilled expectations and related dissatisfaction with contacts with children or friends were more powerful predictors of loneliness among older adults than the frequency of contact with them. Hawkley et al. (2008) further showed that being satisfied with network relationships had an additional protective effect against loneliness. As far as international migrants are concerned, Litwin (1997) showed that after migration the non-kin network types shifted into family-based network types, reflecting a move from a network of choice to a network of necessity on the part of the older immigrants.

#### Meso Level

At the meso level, the community context can provide people with opportunities for community engagement which are important in connectedness and a means of forming new friendships and acquaintances (Fast and De Jong Gierveld 2008; Fraser et al. 2009; Rozanova et al. 2008; Väänänen et al. 2005). Moreover, as Thomése et al. (2003) and Brown et al. (2005) have shown, as mutual concern for the well-being of one’s neighbors and the sense of belonging to the local community increase, the risk of loneliness decreases.

#### Macro Level

Macro-level factors of socio-cultural characteristics of immigrants’ ethnic background and those of the mainstream of the receiving country are not often used in a comparative sense to understand the potential diversity of experiences of older immigrants. Transitions during the life course, especially international migration, have been shown to be decisive in creating opportunities and constraints for older persons to optimize and diversify social contacts in their new environment (Ajrouch et al. 2005; Fokkema et al. 2012; Savikko et al. 2005). For example, Kleinepier (2011) showed immigrants who are disadvantaged in reading and speaking the language of the receiving country have less contact with the native population. Thus it seems likely that lack of language proficiency might then be associated with higher risks of loneliness. Yet the need to belong to one’s country of residence is important: a sense of belonging has been found to be positively associated with feeling positive about oneself (Arredondo 1984; Chow 2007), and negatively associated with feelings of loneliness in later life (De Jong Gierveld et al. 2015).

A few studies have examined the relationship between aging migrants and their social networks and have found that ethnicity and culture, such as differences in values and norms regarding social networks, can influence relationships with kin and non-kin of aging immigrants (Chappell 2003; Dalgard and Thapa 2007; Lai et al. 2007; Mitchell 2003; Rao et al. 2006; Slonim-Nevo et al. 2009). Although kin relationships might be important for all older adults, immigrant adults originating from collectivistic countries might be especially prone to have close family ties that provide (instrumental) support and may alleviate loneliness (Johnson and Mullins 1987; Litwin 1997; Sánchez et al. 2014). Similarly, contacts with friends and participation in clubs and organizations are less valued in collectivistic than individualistic societies (Väänänen et al. 2005; Van Tilburg et al. 1998; Wagner et al. 1999). In this investigation the interrelation between migrant’s ethnic background, social network characteristics and loneliness will explicitly be taken into account.

In addition to the micro, meso and macro contexts described above, several demographic factors known for their effects on loneliness are taken into account in this study. Being married is known to offer the greatest degree of protection against loneliness (De Jong Gierveld et al. 2009), while widowhood and divorce are risk factors (Allen et al. 2000; Dykstra and De Jong Gierveld 2004; Waite and Lehrer 2003). Gender may be important. However, Aartsen and Jylhä (2011) showed that the higher incidence of loneliness among women can be explained by the unequal distribution of the risk of becoming widowed among men and women. Health is important in that those in good health are better positioned to be in contact with social network members. There is considerable evidence that older adults who are in poor health are most prone to high levels of loneliness (De Jong Gierveld et al. 2006; Hawkley et al. 2008; Korporaal et al. 2008; Pinquart and Sörenson 2001; Routasalo et al. 2006; Van Tilburg et al. 2004; Victor et al. 2000).

The evidence to date of relationships of micro, meso and macro factors with loneliness of older adults leads to the following hypotheses: (1) Being an immigrant is associated with higher risks of loneliness; (2) The number of close relatives and close friends, the frequency of contacts, and the satisfaction with these relationships are negatively associated with loneliness; (3) Participation in local organizations, supportive exchanges with neighbours, and a sense of belonging to the local community are negatively associated with loneliness; (4) Immigrants originating from countries with the same mother tongue, and a culture that facilitates contacts with friends born in the host country are less at risk of experiencing loneliness than immigrants who originate from countries with cultural characteristics that are less comparable to the dominant culture of the receiving country.

## Methods

### Respondents

Data for this study were drawn from the public use microdata file of Statistics Canada’s General Social Survey, Cycle 22 (GSS-22) on Social Networks. The GSS-22 sample was selected using Random Digit Dialing and data were collected via Computer Assisted Telephone Interviewing (CATI). Interviews took place from February through November 2008 with 20,401 Canadian men and women aged 15 years or older (excluding full-time residents of institutions and residents of the northern territories). The response rate was 57 %.

Out of the total sample, a sub-sample of 3692 respondents aged 65 years and over who had provided information on loneliness was drawn for the current study. Content relevant to the current study included information on respondents’ migrant status, ethnic-cultural background, contact with friends and relatives, participation in community activities and loneliness.

Given the importance of in-depth knowledge of the language of the receiving country, and the variations in values regarding social relationships, we first differentiate older adults born in Canada from older adults not born in Canada (i.e., those who are immigrants to Canada). Immigrants were further differentiated based on language and cultural background into three subgroups: (1) immigrants of British or French origin, who describe their ethnic background as British or French (7.3 % of respondents); (2) immigrants of non-British or French European origin who describe their ethnic background as other European, such as Germany, the Netherlands and the Ukraine (6.2 % of respondents); or (3) immigrants of non-European origin, who are from world regions other than Europe (5.6 % of respondents). As expected, information about ethnic background is strongly related to the cultural characteristic of first language in childhood. Of the immigrants of British or French origin 97 % mentioned either English or French as first language in childhood. The same figure for immigrants of non-British or French European origin is 10 % and for immigrants of non-European origin 20 %. Interviews were conducted in English or French. Thus those who did not speak either of these languages are not represented in the findings.

### Measurement Instruments

#### De Jong Gierveld Loneliness Scale

The GSS-22 included the short form of the De Jong Gierveld Loneliness Scale, which was the dependent variable in this study. The scale comprises 6 items, 3 of which are indicators of emotional loneliness and 3 are indicators of social loneliness (De Jong Gierveld and Kamphuis 1985; De Jong Gierveld and Van Tilburg 1999, 2006, 2010). An example of the emotional loneliness items is “I experience a general sense of emptiness”. An example of the social loneliness items is “There are enough people I feel close to.” Answer categories are ‘yes’, ‘more or less’ and ‘no’. For details of processing the scale data see De Jong Gierveld and Van Tilburg (1999). The total scores on the loneliness scale ranged from 0, ‘not lonely’, to 6, ‘extremely lonely.’ The scale has been used extensively and found to be reliable and valid as a unidimensional measure of loneliness among older adults (Dykstra and Fokkema 2007; Grygiel et al. 2013; Leung et al. 2008; Pinquart and Sörenson 2001). Moreover, the findings of a study in Canada by Penning et al. (2014) supported the utility of the De Jong Gierveld scale for research involving middle-aged and older adults. Cronbach’s alpha across the 6 items was 0.64 for all respondents in this sample.

#### Control Variables

Demographic and other background characteristics are used as control variables. Sex is a dichotomous variable (male = 0, female = 1) while age is entered as a continuous variable. Health is measured using the direct question: “In general, would you say your health is excellent, very good, good, fair, or poor?” with five answer categories. Marital status is operationalized as a set of dummy variables: married or common law (the reference category in multivariate analyses); widowed (0 = no, 1 = yes); divorced or separated (0 = no, 1 = yes); never married (0 = no, 1 = yes).

#### Micro-Level (Social Context) Variables

There are three kin and three non-kin variables. The first kin variable is frequency of seeing or phoning any of your relatives in the past month. The answer categories ranged from every day to less than once per month. For use in this study answers were operationalized as: weekly or more often = 3, one or more times per month = 2 and less than once per month =1. The second is number of close (intimate) relatives, used as a continuous variable. The third, satisfaction with the frequency of communication with relatives was measured using 5 answer categories ranging from ‘very satisfied’ to ‘very dissatisfied’.

Friend relationships were measured similarly: whether the respondent had any face-to-face and/or telephone contact with friends in the past week or month or less frequently, the number of close friends in the respondent’s network, and satisfaction with the frequency of communication with friends.

#### Meso-Level (Community Context) Variables

There were three community-level variables. Participation in community organisations was measured by the number of organisations in which respondents were involved. Informal involvement in the neighborhood was elicited by two questions: “In the past month, have you done a favor for a neighbor?” and “In the past month have any of your neighbors done a favor for you?” These were operationalized as: both given and received a favor, yes = 1, no = 0. Sense of belonging to the local community was rated on a 5 point scale from ‘very weak’ to ‘very strong.’

#### Macro-Level (Socio-Cultural Embeddedness) Variables

There were two variables in this section. First, the question: “Of all your friends you had contact with in the past month, how many have the same mother tongue as you have?” was used. Answers to this question along with mother tongue are used to create the variable, most or all friends have the same mother tongue not English or French yes = 1, no =0. The second variable is, “Of all your friends you had contact with in the last month, how many came from an ethnic group that is visibly different from yours?” with answer categories ranging from ‘all’ to ‘none’. Responses are coded as: most or all friends are from visibly different group yes = 1, no = 0. Finally, sense of belonging to Canada was used, with answer categories ranging from ‘very weak’ to ‘very strong’.

### Statistical Analysis

Frequencies for categorical variables and means and standard deviations for continuous variables were estimated (Table 1). We used multivariate hierarchical regression models to examine the contribution to variation in loneliness scores of the three sets of variables. Blocks of variables were entered into the models in the following order: (1) micro-level (social context) (2) meso-level (community context), and (3) macro-level (socio-cultural embeddedness). Additionally, interactions between migrant group types and the characteristics at the micro, meso and macro level and loneliness were investigated using UNIANOVA models.

**Table 1:** Sample Characteristics of Canadian Respondents Aged 65 and over (*N* = 3692); Source: Canadian General Social Survey, Cycle 22

	Canadian born (*n* = 2988)	Immigrants of British or French origin (*n* = 269)	Immigrants from Europe (non-British or French) (*n* = 228)	Non-European Immigrants (*n* = 207)	*p*-value*
Loneliness score (0, not lonely → 6, extremely lonely); Mean (SD)	1.26 (1.32)	1.29 (1.38)	1.54 (1.55)	1.93 (1.46)	< 0.001
Micro-level variables					
In the past month did you have at least weekly contact with one of your relatives? % yes	79.9	77.8	75.7	71.3	< 0.05
Number of close relatives; Mean (SD)	7.7 (10.3)	6.0 (5.8)	7.4 (9.3)	6.0 (8.5)	< 0.01
Satisfaction with frequency of contacts with relatives; % very satisfied	93.2	92.1	91.8	92.9	> 0.05
In the past month did you have at least weekly contact with one of your friends? % yes	74.0	81.1	67.8	67.6	< 0.01
Number of close friends; Mean (SD)	6.8 (10.2)	6.9 (8.7)	5.9 (7.5)	6.9 (11.3)	> 0.05
Satisfaction with frequency of contacts with friends; % very satisfied	91.9	90.7	86.8	85.9	< 0.05
Meso-level variables					
Member or participant in groups or organisations? % yes	60.0	62.5	47.6	47.6	< 0.001
In the past month, have you done a favor for a neighbor and have any of your neighbors done a favor for you? % both gave and received	51.9	57.6	41.4	38.1	< 0.01
Sense of belonging to your local community; % very strong	34.2	32.9	29.4	27.3	< 0.01
Macro-level variables					
If mother tongue is not-British or French: of the friends you had contact with in the past month, how many have the same mother tongue as you? % same mother tongue	3.4	1.9	38.7	48.1	< 0.001
All or most friends from an ethnic group visibly different from yours? % yes	4.4	6.3	10.7	17.3	< 0.001
Sense of belonging to Canada; % very strong	73.0	77.8	78.6	77.3	> 0.05
Control variables					
Sex; % female	55.2	57.9	54.1	51.6	> 0.05
Age					
65–69	33.6	23.1	29.0	32.2	> 0.05
70–74	25.4	21.1	25.3	24.3	
75–79	18.6	24.7	20.5	20.8	
80 +	22.5	31.2	25.3	22.7	
Subjective health: % fair or poor	23.6	23.8	35.5	34.1	< 0.001
Widowed? % yes	24.3	23.2	18.9	22.8	> 0.05
Divorced or separated? % yes	7.1	7.2	10.1	6.3	> 0.05
Never married? % yes	4.1	2.4	1.6	3.6	> 0.05

Weighted percentages, unweighted N. SD: standard deviation

*p-value for a chi-square or *F*-test

## Results

### Descriptive Statistics

Descriptive statistics for the dependent and independent variables for older adults in each of the immigrant and the non-immigrant groups can be found in Table 1. Loneliness mean score for the total sample is 1.32 (*SD* = 1.36). Those born in Canada score a mean of 1.26 (SD = 1.32). Mean loneliness for all immigrant groups is significantly higher at 1.55 (mean difference = 0.29, *SE* = 0.09, *p* < 0.05). However, there were significant differences among the three groups of immigrants. There is no significant difference in loneliness between immigrants of British or French ethnic origin (M = 1.29) and respondents born in Canada (Mean difference =0.03, *SE* = 0.09, *p* = 0.993). In contrast, loneliness is significantly higher for immigrants with other European origin (M = 1.54) compared to those born in Canada (mean difference = 0.28, *SE* = *0*.09, *p* < 0.05). Finally, non-European immigrants have the highest loneliness scores (M = 1.93), significantly higher than loneliness of those born in Canada (mean difference = 0.66, *SE* = 0.10, *p* < 0.001).

Descriptive results on demographic (control) and context variables also are shown in Table 1. Among demographic variables, the four groups are fairly evenly distributed across gender, age and marital status. However, other European (non-British or French) and non-European immigrants have significantly poorer subjective health in comparison to Canadian born respondents and immigrants of British or French origin.

This relative lack of personal resources of these two immigrant groups from places that do not share Canadian language and culture is reflected across micro, meso and macro contexts. At the micro level (social context variables), non-European immigrants see and/or phone relatives less frequently than those in other groups. Friend networks differ as well. Immigrants from other parts of Europe (non-British or French) and non-European immigrants see and/or phone friends less frequently than either Canada born or immigrants of British or French origin. Immigrants from Europe (non-British or French) and of non-European origin are significantly less satisfied with the frequency of communication with their friends compared to other respondents. An exception to this pattern is immigrants of British or French origin who have the fewest close relatives.

At the meso level (community variables), immigrants from Europe (non-British or French) and non-European immigrants are significantly less involved in the local community; that is, they more frequently have no organizational memberships, are not involved in support exchanges with neighbors, and have a lower sense of belonging to the local community compared to immigrants from Europe (British or French origin) and those who are Canadian born.

At the macro level (socio-cultural embeddedness variables), Canadian born and immigrants from Europe (British or French origin) are significantly more likely to be surrounded by friends with predominantly the same mother tongue. More than 80 % of these respondents mentioned that most or all of their friends have the same mother tongue while fewer than 10 % had all or most of their friends from an ethnic group visibly different from their own. In contrast, older European (non-British or French origin) immigrants and those of non-European origin are characterized by a substantially different composition of non-kin networks. Approximately 50 to 60 % of their friends have another mother tongue and 10 to 20 % have all or most friends who are visibly different from themselves. In terms of the sense of belonging to Canada, more than 70 % of each group expressed a (very) strong sense of belonging to Canada—with the lowest proportion those who were Canadian born.

Together these findings provide an initial picture of immigrants from Europe (non-British or French) and non-European immigrants as lonelier and less embedded in social and community networks than Canadian born respondents or immigrants of British or French origin.

### Multivariate Analysis

Table 2 displays the results of the multivariate hierarchical regression analysis of loneliness on micro-, meso- and macro-level variables.

**Table 2:** Summary of hierarchical regression analysis for variables predicting loneliness; Canadian men and women aged 65 and over (Source GSS-cycle 22; *N* = 3692)

Variables	Model 1			Model 2			Model 3			Model 4		
	B	SE	β	B	SE	β	B	SE	β	B	SE	β
Constant	0.527			0.360			0.866			1.064		
Canadian born (ref)												
Immigrant, British/French origin	0.06	0.08	0.01	0.03	0.08	0.01	0.02	0.08	0.00	0.03	0.08	0.01
Immigrant, Europe (non British/French)	0.22	0.09	0.04*	0.20	0.09	0.04 *	0.16	0.09	0.03	0.10	0.09	0.02
Immigrant Non-European	0.63	0.09	0.11***	0.57	0.09	0.10***	0.54	0.09	0.09***	0.44	0.10	0.08***
*Micro*-*level variables* Frequency contact with relatives; less than monthly → weekly				0.02	0.04	0.01	0.04	0.04	0.02	0.04	0.04	0.01
Number close relatives				−0.01	0.00	−0.09***	−0.01	0.00	−0.09***	−0.01	0.00	−0.09***
Satisfaction freq contact w. relatives; very satisfied → not very satisfied				0.33	0.03	0.19***	0.31	0.03	0.18***	0.31	0.03	0.18***
Frequency contact with friends; less than monthly → weekly				-0.18	0.03	−0.09***	−0.13	0.03	−0.07***	−0.14	0.03	−0.07***
Number close friends				−0.01	0.00	−0.06***	−0.01	0.00	−0.05**	−0.01	0.00	−0.05**
Satisfaction freq. contacts w. friends; very satisfied → not very satisfied				0.21	0.03	0.11***	0.19	0.03	0.10***	0.19	0.03	0.10***
*Meso*-*level variables* Member/participant in groups; 4 or more → none							0.04	0.04	0.03	0.03	0.02	0.02
Favor for neighbor given and received; no-yes							−0.06	0.04	−0.02	−0.06	0.04	−0.02
Sense of belonging to local community: very weak→ very strong							−0.22	0.03	−0.12***	−0.21	0.03	−0.12***
*Macro*-*level variables* Mother tongue not English/French & most friends same mother tongue; no-yes										0.21	0.09	0.04*
All or most friends from an ethnic group visibly different from yours? no-yes										0.08	0.09	0.01
Sense of belonging to Canada; Very weak → very strong										−0.04	0.03	−0.02
Control variables												
Sex; M-F	−0.10	0.05	−0.04*	−0.06	0.05	−0.02	−0.06	0.05	−0.02	−0.06	0.05	−0.02
Subj. health: excellent →poor	0.23	0.02	0.22***	0.21	0.02	0.17***	0.20	0.02	0.16***	0.20	0.02	0.16***
Married/ common law (ref)												
Widowed	0.25	0.05	0.09***	0.26	0.05	0.09***	0.25	0.05	0.09***	0.25	0.05	0.09***
Divorced/separated	0.39	0.07	0.09***	0.30	0.07	0.07***	0.24	0.07	0.05**	0.24	0.07	0.06**
Never married	0.23	0.10	0.04*	0.24	0.10	0.04*	0.20	0.10	0.03*	0.20	0.10	0.03*
R^2^ adj.	0.074			0.158			0.173			0.175		

* *p* < 0.05; ** *p* < 0.01; ***p < 0.001

Model 1shows that, after controlling for demographic variables and health, the three immigrant subgroups differ in loneliness outcomes. Immigrants from Europe of British or French origin are not significantly different from those born in Canada. However, immigrants from other parts of Europe (not British or French) and non-European origin are significantly lonelier. Variance explained is 7.4 %.

In model 2, micro (social context) variables were entered. Of the six variables related to networks of relatives and friends, five were significant. Number of close relatives and satisfaction with the frequency of contacts with relatives are negatively related to loneliness. Frequency of contact with friends, number of close friends and satisfaction with the frequency of contact with friends are negatively related to loneliness. When this block of social context variables was entered, immigrants from other European countries (non-British or French) and non-European immigrants continued to be significantly lonelier than Canadian born participants. Variance explained is 15.8 %.

In model 3, meso (community context) variables were entered. Of the three variables, only stronger sense of belonging to the local community was significantly related to loneliness. Introducing community level variables mediated the relationship between immigrant subgroups and loneliness. The association between loneliness and immigrants from other parts of Europe (non-British or French) is no longer significant. Variance explained is 17.3 %.

In model 4, macro (socio-cultural embeddedness) variables were entered. One of the three variables is significant. Having most friends who do not speak English or French as their mother tongue is significantly and positively associated with loneliness. Immigrants of non-European origin continue to be significantly lonelier than other immigrant groups. Total variance explained by model 4 is 17.5 %.

In investigating interactions among immigrant groups, kin relationships and loneliness, no significant moderators were found. There were two significant moderators regarding friendships. For non-European immigrants both the number of close friends and satisfaction with contact with friends were inversely related loneliness, but not to the same extent as for other immigrant subgroups. There also were two significant moderators regarding community participation. Non-European migrants if less involved in local organizations and clubs, are lonelier but not to the same extent as other immigrant groups. Further, immigrants from other parts of Europe (non-British or French origins) and non-European immigrants, who are characterized by a stronger sense of belonging to the new home country are less lonely, but not to the same extent as immigrants of British or French origin or those who are Canadian born. In model 4, none of the interactions were significant.

## Discussion

This study supports previous findings about immigration as a risk factor for negative outcomes in later life (Victor et al. 2012; Wu and Penning 2015). Our results showed that mean loneliness of older immigrants to Canada is significantly higher than that of older adults who are Canadian-born. Yet we also have highlighted diversity among immigrants, finding significant differences in the intensity of loneliness among immigrant subgroups. Immigrants who share both the native language of the receiving country and a similar culture do not differ from native born older adults. Those who share neither have significantly higher mean loneliness scores. Thus Hypothesis 1 that immigration is associated with higher risks of loneliness, is supported only at the aggregate level. Future research into immigrants’ loneliness needs to take into account the ethnic-cultural background both of immigrants to the particular country and of the match between their language and culture and that of the receiving country.

Results from this study provide insight into the contributions of micro-level social network resources, meso-level involvement in the local community, and macro-level socio-cultural embeddedness to our understanding of loneliness. We highlight themes from our findings and comment on the relative advantages and limitations of using an ecological approach to examine contexts relevant to older immigrants.

Our second hypothesis was that the social context of relatives and friends is associated with the intensity of loneliness. For the most part, this hypothesis was supported. Numbers of network members, contact with them and satisfaction with contact all are associated with loneliness. The social contexts add considerably to the explained variance in our hierarchical regression model and findings are robust across models in which other contexts are entered.

There is one notable exception to these findings. Frequency of contact with relatives was not significant. This finding warrants further exploration in light of evidence of ambivalence in families that suggests that contact may not always be positive given the potential for tensions within close family groups (Pillemer et al. 2012). The lack of significant relationships between family contact and loneliness also may result from incongruities between relationship standards and experiences for some immigrant groups whose values related to filial piety and intergenerational households differ from those of household independence and intimacy at a distance in countries such as Canada (Rochelle et al. 2009; Treas and Mazumdar 2002). Additionally, the outcomes of the multivariate analyses showed a significant interaction term regarding friendship relationships. Effects of close friendships and satisfaction with contacts with friends moderate the relationship between friendship characteristics and loneliness for non-European immigrants. It seems that non-family relationships and frequent contact with friends are less salient among this group of immigrants of non-European origin.

The meso-level community context was less important in understanding loneliness. Neither participation in local organizations and clubs, nor having supportive relationships with neighbours, was significantly associated with loneliness. Thus hypothesis 3, that community participation is negatively associated with loneliness, was not supported. This finding is similar to that of Ip et al. (2007) who found that non-European immigrants residing in Australia, are characterized by locally restricted activity patterns that were typified by relatively little participation in the broader local community. In our study, we found a significant interaction effect related to community participation. Non-European immigrants who were less involved in local organizations and clubs were lonelier but not to the same extent as other migrant groups, suggesting that active community participation is not necessarily an important goal for all.

Sense of belonging to the local community was significantly related to loneliness, suggesting that there may be different pathways to sense of belonging than through community participation. In general, these findings suggest that there is still much to learn about diversity in immigrants’ socio-cultural embeddedness in later life.

At the macro level (socio-cultural embeddedness), hypothesis 4 was partially supported. Having a network of people who speak your native language is significantly associated with higher risks of loneliness. It may be that while such connections are comforting, they keep people focused on culture lost, precluding a sense of strong local embeddedness. Or it may be that such networks are very small and lack the social capital to help connect this immigrant to a broader network of people across a broader geographic space. Of the three contexts investigated in this study, we know least about how the macro context might influence loneliness of older immigrants.

Overall, the ecological approach used in this study to frame our exploration of contexts relevant to loneliness of older immigrants has resulted in insights into the determinants of loneliness at several levels of the ecological system. The approach also has prompted us to consider more explicitly in future work an additional human ecology assumption that may help us address questions raised in this research. For example, a basic premise of the frameworks is that boundaries between contexts are permeable so that characteristics of one interact with and can influence others (Keating and Phillips 2008). For example, the ethnic mix in the meso (community) context may influence the opportunities for the development of friend networks in the meso (social) context. Understanding this interaction may be especially important in determining interventions to alleviate intense loneliness experienced by immigrants who are unfamiliar with local language or culture.

A second human ecology assumption is that of the importance of person-environment fit (Scheidt and Norris-Baker 2004). The idea is that the best outcomes occur when there is a good fit between personal resources and contexts in which people are living. One might argue that Canadian born and immigrants of British or French origin have lowest levels of loneliness because their language and culture are congruent with the contexts in which they live. Similarly, those who lack these similarities in language or culture are less likely to experience a comfortable fit between their backgrounds and their living contexts. From this perspective, loneliness is an outcome of a poor fit between person and context.

The framework used here addresses a call for better theorizing about ageing and immigration. McDonald (2011) has argued that complex issues of both ageing and of immigration require an integrative framework with multiple levels and a lifecourse perspective. Lifecourse issues may be particularly important in developing further understanding of the experiences of aging immigrants. In this study we were not able to address the timing of the migration transition. Many immigrants have aged in Canada, but there are also those who have immigrated as older persons, having spent most of their life course in their country of origin (Durst 2005). Age of immigration, length of time in the new country or reasons for migration, all might influence the person-environment fit experienced in later life. Future research might focus on variations in social integration and loneliness among naturalized citizens, landed immigrants, refugees, and non-permanent residents, taking into account age of arrival.

### Limitations

Secondary analyses of large national data sets have considerable advantage in terms of national representativeness but also limitations as were evident with this study. First, in this study we created three broadly defined groups of immigrants based on similarity of language and culture to Canada. This broad categorization has been useful in challenging the notion of similarity across immigrants’ experiences. However, it does not capture well the potential heterogeneity of specific ethnic groups. Further exploration of the relative importance of having language or culture similar to that of the receiving country would have been facilitated had we been able to define groups of immigrants who for example came from countries in which the first language is French, but the culture is different from Canada. African countries such as Congo or Cameroon are examples. Such differentiation was not possible due to the relatively small numbers of older immigrant respondents from some countries.

More information on reasons for immigration also might have added to our understanding of risk factors for loneliness. For example, some immigrants come to Canada as part of a family reunification program in which spouses, children and parents of Canadian citizens or permanent residents are given priority. Such immigrants might be more connected to relatives than those who come as refugees, often leaving behind others who are close kin. Such questions were not asked in this study.

In the General Social Survey series from which data for this study were drawn, Statistics Canada conducts interviews in either English or French. This procedure increases the likelihood that immigrants who are not fluent in one of these languages will not participate. Such respondents are most likely to be in the group we designated as ‘non-European’ and who are at highest risk of loneliness. It seems likely that they are underrepresented in this sample, perhaps masking the depth or prevalence of loneliness among them.

Finally, in investigating the relationship between network of kin relationships, and loneliness, we were not able to determine the effects of particular relationships such as those with children since specific kin ties were not addressed separately in the questionnaire. Given the importance of adult children for the well-being of older adults, this has hindered a more in-depth analysis of an important aspect of family life.

As the numbers of aging immigrants further increase in Canada, we must recognize the increasing diversity among ethnic/cultural groups. Policy makers have to keep in mind that immigration to Canada does not necessarily lead to becoming embedded in social networks of kin and non-kin, in the social fabric of the local community, or in an identity that results from experiencing a new cultural context (see also Kim et al. 2012). The negative experience of an immigrant whose social network is unsupportive, who experiences less than desired involvement in local activities and who faces challenges in adapting to the dominant host culture puts them at greater risks of loneliness. Knowledge about the determinants of loneliness is a first requirement to enable policy makers and others to better assess the needs of Canada’s aging immigrant population and to suggest policies that will support its increasingly diverse aging population.

## References

[CR1] Aartsen, M. J., & Jylhä, M. (2011). Onset of loneliness in older adults: results of a 28 year prospective study. *European Journal of Ageing, 8*(1), 31–38. 10.1007/s10433-011-0175-7 PMC3047676 21475393

[CR2] Ajrouch K, Blandon AY, Antonucci TC (2005). Social networks among men and women: the effects of age and socioeconomic status. Journal of Gerontology: Social Sciences.

[CR3] Allen KR, Blieszner R, Roberto KA (2000). Families in the middle and later years: a review and critique of research in the 1990s. Journal of Marriage and the Family.

[CR4] Arredondo PM (1984). Identity themes for immigrant young adults. Adolescence.

[CR5] Blieszner R, Adams RG (1992). Adult friendship.

[CR6] Boyd M, Vickers M (2000). 100 years of immigration in Canada. Canadian Social Trends Statistics Canada.

[CR7] Bronfenbrenner U (1994). Ecological models of human development, International encyclopedia of education, vol. 3.

[CR8] Brown WM, Consedine NS, Magai C (2005). Altruism relates to health in an ethnically diverse sample of older adults. Journal of Gerontology: Psychological Sciences.

[CR9] Cacioppo JT, Patrick W (2009). Loneliness: Human nature and the need for social connection.

[CR10] Castles S, de Haas H, Miller MJ (2014). The age of migration: International population movements in the modern world.

[CR11] Chappell NL (2003). Correcting cross-cultural stereotypes: aging in Shanghai and Canada. Journal of Cross-Cultural Gerontology.

[CR12] Chow HPH (2007). Sense of belonging and life satisfaction among Hong Kong adolescent immigrants in Canada. Journal of Ethnic and Migration Studies.

[CR13] Dalgard OS, Thapa SB (2007). Immigration, social integration and mental health in Norway, with focus on gender differences. Clinical Practice and Epidemiology in Mental Health.

[CR14] De Haas H, Fokkema T (2010). Intra-household conflicts in migration decision-making: return and pendulum migration in Morocco. Population and Development Review.

[CR15] De Jong Gierveld J (1987). Developing and testing a model of loneliness. Journal of Personality and Social Psychology.

[CR16] De Jong Gierveld J, Kamphuis F (1985). The development of a Rasch-type loneliness scale. Applied Psychological Measurement.

[CR17] De Jong Gierveld J, Van Tilburg TG (1999). Manual of the loneliness scale.

[CR18] De Jong Gierveld J, Van Tilburg T (2006). A six-item scale for overall, emotional and social loneliness: confirmative tests on new survey data. Research on Aging.

[CR19] De Jong Gierveld J, Van Tilburg T (2010). The De jong gierveld short scales for emotional and social loneliness: tested on data from seven countries in the UN generations and gender surveys. European Journal of Ageing.

[CR20] De Jong Gierveld J, Van Tilburg T, Dykstra PA, Vangelisti A, Perlman D (2006). Loneliness and social isolation. Cambridge handbook of personal relationships.

[CR21] De Jong Gierveld J, Broese van Groenou M, Hoogendoorn AW, Smit JH (2009). Quality of marriages in later life and emotional and social loneliness. The Journals of Gerontology Series B: Psychological Sciences and Social Sciences.

[CR22] De Jong Gierveld J, Keating N, Fast J (2015). Determinants of loneliness among older adults in Canada. Canadian Journal of Aging.

[CR23] Durst D (2005). Aging amongst immigrants in Canada: population drift. Canadian Studies in Population.

[CR24] Dykstra PA (1993). The differential availability of relationships and the provision and effectiveness of support to older adults. Journal of Social and Personal Relationships.

[CR25] Dykstra PA, De Jong Gierveld J (2004). Gender and marital-history differences in emotional and social loneliness among Dutch older adults. Canadian Journal of Aging.

[CR26] Dykstra PA, Fokkema T (2007). Social and emotional loneliness among divorced and married men and women: comparing the deficit and cognitive perspectives. Basic and Applied Social Psychology.

[CR27] Campaign to End Loneliness (2011). http://www.campaigntoendloneliness.org/ Accessed 12 Dec 2014.

[CR28] Fast JE, De Jong Gierveld J, Keating NC (2008). Ageing, disability and participation. Rural Ageing: A good place to grow old?.

[CR29] Fokkema T, De Jong Gierveld J, Dykstra PA (2012). Cross-national differences in older adult loneliness. The Journal of Psychology.

[CR30] Fraser J, Clayton S, Sickler J, Taylor A (2009). Belonging to the zoo: retired volunteers, conservation activism and collective identity. Ageing and Society.

[CR31] George U, Westhues A (2006). Immigration and refugee policy in Canada: Past, present and future. Canadian social policy.

[CR32] Grygiel P, Humenny G, Rebisz S, Świtaj P, Sikorska J (2013). Validating the polish adaptation of the 11-item De Jong Gierveld loneliness scale. European Journal of Psychological Assessment.

[CR33] Hawkley LC, Hughes ME, Waite LJ, Masi CM, Thisted RA, Cacioppo JT (2008). From social structural factors to perceptions of relationship quality and loneliness: the Chicago health, aging, and social relations study. Journal of Gerontology Social Sciences.

[CR34] Ip D, Lui CW, Chui WH (2007). Veiled entrapment: a study of social isolation of older Chinese migrants in Brisbane, Queensland. Ageing and Society.

[CR35] Johnson DP, Mullins LC (1987). Growing old and lonely in different societies: toward a comparative perspective. Journal of Cross-Cultural Gerontology.

[CR36] Kalache, A. (2013). Responding to the longevity revolution worldwide. Keynote presentation to the Sheridan Elder Research Centre (SERC).from https://www.sheridancollege.ca/news-and-events/news/alexandre-kalache-uses-wisdom-and-humour-to-raise-awareness-about-global-aging.aspx. Accessed 30 Oct 2014

[CR37] Keating N, Phillips JE, Keating N (2008). A critical human ecology perspective on rural ageing. Rural ageing: A good place to grow old?.

[CR38] Keating N, Scharf T, Scharf T, Keating N (2012). Revisiting social exclusion of older adults. From exclusion to inclusion in old age: A global challenge.

[CR39] Kim BJ, Sangalang CC, Kihl T (2012). Effects of acculturation and social network support on depression among elderly Korean immigrants. Aging & Mental Health.

[CR40] Kleinepier T (2011). Age at immigration and second language proficiency among immigrants in the Netherlands; Maturational constraints or life-course trajectories? unpublished empirical research.

[CR41] Korporaal M, Broese van Groenou M, Van Tilburg TG (2008). Effects of own and spousal disability on loneliness among older adults. Journal of Aging and Health.

[CR42] Lai DWL, Tsang KT, Chappell NL, Lai DCY, Chau SBY (2007). Relations between culture and health status: a multi-site study on older Chinese in Canada. Canadian Journal of Aging.

[CR43] Leung GTY, De Jong Gierveld J, Lam LCW (2008). Validation of the Chinese translation of the 6-item De Jong Gierveld loneliness scale in elderly Chinese. International Psychogeriatrics.

[CR44] Lewis DC (2009). Aging out of place: Cambodian refugee elders in the United States. Family and Consumer Sciences Research Journal.

[CR45] Litwin H (1997). The network shifts of elderly immigrants: the case of soviet Jews in Israel. Journal of Cross-Cultural Gerontology.

[CR46] McDonald L (2011). Theorising about ageing, family and immigration. Ageing and Society.

[CR47] Mitchell BA (2003). Would I share a home with an elderly parent? Exploring ethnocultural diversity and intergenerational support relations during young adulthood. Canadian Journal of Aging.

[CR48] Newall N, Chipperfield J, Bailis D (2014). Predicting stability and change in loneliness in later life. Journal of Social and Personal Relationships.

[CR49] Penning MJ, Liu G, Chou PHB (2014). Measuring loneliness among middle-aged and older adults: the UCLA and de Jong Gierveld loneliness scales. Social Indicator Research.

[CR50] Perlman D, Peplau LA, Gilmour R, Duck S (1981). Toward a social psychology of loneliness. Personal relationships 3: Personal relationships in disorder.

[CR51] Pillemer K, Munsch CL, Fuller-Rowell T, Riffin C, Suitor J (2012). Ambivalence toward adult children: differences between mothers and fathers. Journal of Marriage and Family.

[CR52] Pinquart M (2003). Loneliness in married, widowed, divorced, and never-married older adults. Journal of Social and Personal Relationships.

[CR53] Pinquart M, Sörenson S (2001). Gender differences in self-concept and psychological well-being in old age: a meta-analysis. Journal of Gerontology: Psychological Sciences.

[CR54] Rao D, Warburton J, Bartlett H (2006). Health and social needs of older Australians from culturally and linguistically diverse backgrounds: Issues and implications. Australasian Journal on Ageing.

[CR55] Rochelle TL, Shardlow SM, Ng SH (2009). Ageing in Hong Kong and the UK: a comparison of the Chinese at home and abroad. Journal of Comparative Asian Development.

[CR56] Routasalo PE, Savikko N, Tilvis RS, Strandberg TE, Pitkala KH (2006). Social contacts and their relationships to loneliness among aged people: a population-based study. Gerontology.

[CR57] Rozanova J, Dosman D, De Jong Gierveld J, Keating NC (2008). Participation in rural contexts: community matters. Rural Ageing: A good place to grow old?.

[CR58] Sánchez MM, de Jong Gierveld J, Buz J (2014). Loneliness and the exchange of social support among older adults in Spain and the Netherlands. Ageing and Society.

[CR59] Savikko N, Routasalo PE, Tilvis RS, Strandberg TE, Pitkala KH (2005). Predictors and subjective causes of loneliness in an aged population. Archives of Gerontology and Geriatrics.

[CR60] Scharf T, Keating N, Scharf T, Keating N (2012). Social exclusion in later life: A global challenge. From exclusion to inclusion in old age.

[CR61] Scheidt RJ, Norris-Baker C, Wahl H-W, Scheidt R, Windley PG (2004). The general ecological model revisited: Evolution, current status, and continuing challenges. Annual review of gerontology and geriatrics: Aging in context: socio-physical environments.

[CR62] Slonim-Nevo V, Rubinstein J, Nauck B (2009). The impact of familial and environmental factors on the adjustment of immigrants: a longitudinal study. Journal of Family Issues.

[CR63] Statistics Canada. (2011a). *2006 Census of Population (Catalogue No. 97-57-XCB2006015)*. Ottawa: Statistics Canada.

[CR64] Statistics Canada (2011b). 2011 National Household Survey: Data tables. Calculated from http://www12.statcan.gc.ca/nhs-enm/2011/dp-pd/dt-td/Lp-eng.cfm?LANG=E&APATH=3&DETAIL=0&DIM=0&FL=A&FREE=0&GC=0&GID=0&GK=0&GRP=0&PID=0&PRID=0&PTYPE=105277&S=0&SHOWALL=0&SUB=0&Temporal=2013&THEME=95&VID=0&VNAMEE=&VNAMEF

[CR65] Thomése F, van Tilburg T, Knipscheer CPM (2003). Continuation of exchange with neighbors in later life: the importance of the neighborhood context. Personal Relationships.

[CR66] Treas J, Batlova J, Uhlenberg P (2009). Immigrants and aging. International handbook of population aging.

[CR67] Treas J, Mazumdar S (2002). Older people in America’s immigrant families: dilemmas of dependence, integration, and isolation. Journal of Aging Studies.

[CR68] Väänänen A, Buunk BP, Kivimäki M, Pentti J, Vahtera J (2005). When it is better to give than to receive?: Long-term health effects of perceived reciprocity in support exchange. Journal of Personality and Social Psychology.

[CR69] Van Tilburg T, de Jong Gierveld J, Lecchini L, Marsiglia D (1998). Social integration and loneliness: a comparative study among older adults in the Netherlands and Tuscany, Italy. Journal of Social and Personal Relationships.

[CR70] Van Tilburg T, Havens B, De Jong Gierveld J (2004). Loneliness among older adults in the Netherlands, Italy, and Canada: a multifaceted comparison. Canadian Journal of Aging/La Revue canadienne du vieillissement.

[CR71] Victor C, Scambler S, Bond J, Bowling A (2000). Being alone in later life: loneliness, social isolation and living alone. Reviews in Clinical Gerontology.

[CR72] Victor C, Burholt V, Martin W (2012). Loneliness and ethnic minority elders in Great Britain: an exploratory study. Journal of Cross-Cultural Gerontology.

[CR73] Viruell-Fuentes E, Miranda P, Abdulrahim S (2012). More than culture: structural racism, intersectionality theory, and immigrant health. Social Science and Medicine.

[CR74] Voorpostel M, Van der Lippe T (2007). Support between siblings and between friends: two worlds apart?. Journal of Marriage and Family.

[CR75] Wagner M, Schütze Y, Lang FR, Baltes PB, Mayer KU (1999). Social relationships in old age. The Berlin aging study: aging from 70 to 100.

[CR76] Waite LJ, Lehrer EL (2003). The benefits from marriage and religion in the United States: a comparative analysis. Population and Development Review.

[CR77] Walker A (2009). Commentary: the emergence and application of active aging in Europe. Journal of Aging & Social Policy.

[CR78] Wong S, Yoo G, Stewart A (2005). Examining the types of social support and the actual sources of support in older Chinese and Korean immigrants. The International Journal of Aging and Human Development.

[CR79] Wu Z, Penning M (2015). Immigration and loneliness in later life. Ageing and Society.

